# Making sense of COVID-19 over time in New Zealand: Assessing the public conversation using Twitter

**DOI:** 10.1371/journal.pone.0259882

**Published:** 2021-12-15

**Authors:** Hamed Jafarzadeh, David J. Pauleen, Ehsan Abedin, Kasuni Weerasinghe, Nazim Taskin, Mustafa Coskun

**Affiliations:** 1 School of Management, Massey Business School, Massey University, Auckland, New Zealand; 2 School of Computing and Information Systems, The University of Melbourne, Melbourne, Australia; 3 Department of Management Information Systems, Bogazici University, Istanbul, Turkey; 4 Information Technologies Department, Bornova Science and Art Center, Ministry of National Education, Izmir, Turkey; King Abdulaziz University, SAUDI ARABIA

## Abstract

COVID-19 has ruptured routines and caused breakdowns in what had been conventional practice and custom: everything from going to work and school and shopping in the supermarket to socializing with friends and taking holidays. Nonetheless, COVID-19 does provide an opportunity to study how people make sense of radically changing circumstances over time. In this paper we demonstrate how Twitter affords this opportunity by providing data in real time, and over time. In the present research, we collect a large pool of COVID-19 related tweets posted by New Zealanders–citizens of a country successful in containing the coronavirus–from the moment COVID-19 became evident to the world in the last days of 2019 until 19 August 2020. We undertake topic modeling on the tweets to foster understanding and sensemaking of the COVID-19 tweet landscape in New Zealand and its temporal development and evolution over time. This information can be valuable for those interested in how people react to emergent events, including researchers, governments, and policy makers.

## 1. Introduction

COVID-19 has swept across the world, disrupting individuals and communities, challenging people’s assumptions about safety and security, and in general causing physical and psychological upheaval [1], around issues such as health, elderly or vulnerable family members, travel restrictions, job losses, school closures, economic impacts, etc. [2]. When emergent complex events such as COVID-19 occur, those affected grasp for meaning and stability [2]. Without the usual frames of reference for personal and social behavior, how do they achieve equilibrium amid the chaos?

COVID-19 provides an opportunity to study how people make sense of radically changing circumstances, and social media affords this opportunity by providing data in real time, and over time. In recent years, especially after the arrival of COVID-19, a growing steam of studies have emerged in which researchers use big data analytics to understand and make sense of general public discourse about epidemic diseases as reflected on social media, including, for example, tracking rapidly evolving public conversation and sentiments, gauging public interests and concerns, uncovering real-time disease conversational trends, and so on [3]. Table 1 provides a summary of such studies across various topics, use-cases, and regions/countries. The present paper contributes to this stream of research by looking at one country, New Zealand, with a relatively homogenous citizenry and a government that has been generally recognized to have proactively engaged with the COVID-19 event and contained the virus successfully [4, 5]. Our interest is to understand the process of how the people of New Zealand came to understand the emergence and potential threat of COVID-19 and how they reacted to the subsequent effects. To do this, we track and analyze tweets from New Zealanders from the moment COVID-19 became evident to the world in late December 2019 through successive key dates in New Zealand. These included progressively restrictive lockdowns to a state of total lockdown to progressively relaxed lockdowns and then back up as a second wave of the virus appeared in mid-August 2020. We map Twitter data through topic modeling to explore how the New Zealand public (Twitter users) understood and made sense of the COVID-19 pandemic and its effects on individuals and society during this time.

**Table 1:** Sample of topic modeling studies to make sense of tweets about epidemic disease.

Study		Disease		Region studied		# of tweets and time duration		Brief description/findings	
Xue, et al. [3]		COVID-19		Worldwide		1.9 million tweets (23 Jan to 7 Mar 2020)		Analyzed tweets related to COVID-19 fetched through 19 trending hashtags. Identified 11 topics.	
Lyu and Luli [22]		COVID-19		USA		290,764 tweets (11 Mar 2020 to 14 Aug 2020)		Identified the topics and their overarching themes emerging from the public COVID-19-related tweet discussion about the Centers for Disease Control and Prevention in USA	
Massaro, et al. [17]		COVID-19		Italy		74,306 tweets (11 Feb to 10 Mar 2020)		Highlighted critical dimensions of conversations around COVID-19. Findings underlined the importance of social media platforms in engagement and gaining the public’s trust during a pandemic.	
Jang, et al. [28]		COVID-19		North America		319,524 tweets (21 Jan to 11 May)		Explored people’s concerns and reactions to COVID-19 in North America, particularly in Canada via analyzing COVID-19 related tweets	
de Melo and Figueiredo [25]		COVID-19		Brazil		1,597,934 tweets (Jan to May 2020)		Captured the main subjects and themes under discussion in social media (and news media) to analyze the impact of the COVID-19 pandemic in Brazil.	
Saleh, et al. [29]		COVID-19		Worldwide		574,903 tweets (27 Mar to 10 Apr 2020)		Examined public perception of social distancing through organic, large-scale discussion on Twitter.	
Alshalan, et al. [27]		COVID-19		Arab region		975,316 tweets (27 Jan to 30 Apr 2020)		Identified hate speech related to the COVID-19 pandemic posted by Twitter users in the Arab region to discover the main issues discussed.	
Wang, et al. [26]		COVID-19		China		203,191 tweets (1 Dec 2019 to 30 Jul 2020)		Examined the main concerns raised and discussed by citizens on Sina Weibo, the largest social media platform in China, during the COVID-19 pandemic (a none-Tweeter study).	
Boon-Itt and Skunkan [30]		H1N1 Outbreak		Worldwide		2 million tweets (1 May to 31 December 2019)		Illustrates the potential of using social media to conduct ‘‘infodemiology” studies for public health. Showed that Tweets can be used for real-time content analysis and knowledge translation research, allowing health authorities to respond to public concerns.	
Wicke and Bolognesi [31]		COVID-19		Worldwide		203,756 tweets (20 Mar 30 April 2020)		Showed a plethora of framing options—or a metaphor menu—may facilitate the communication of various aspects involved in the COVID-19 related discourse on the Twitter, and thus support civilians in the expression of their feelings, opinions and beliefs during the pandemic.	
Li, et al. [24]		COVID-19		USA		80 million tweets (January 2020 to April 2020)		Detected stress symptoms related to COVID-19 in the United States. The results reveal a strong correlation between stress symptoms and the number of increased COVID-19 cases for major U.S. cities	
Liu, et al. [32]		COVID-19		China		Articles from WiseSearch		Extracted twenty topics and then classified them into nine themes. The topics include medical affiliation and staff, prevention and control policy, epidemiologic study, etc. The themes include prevention and control procedures, detection on public transportation, confirmed cases, medical treatment and research, etc.	
Abd-Alrazaq, et al. [33]		COVID-19		Worldwide		167,073 tweets (2 February 2020 to 15 March 2020)		Using topic modeling, extracted four themes of: the source of COVID-19, the origin of COVID-19, the impact of COVID-19 on countries and people, and the methods for decreasing the spread of COVID-19	
Kaila, et al.[34]		COVID-19		Worldwide		18000 tweets		Extracted the topics from tweets related to COVID-19	
Shorey, et al. [35]		COVID-19		Singapore		2075 comments of 29 local Facebook news articles (23 January 2020 to the 3 April 2020)		Realized that concern and fear were the main reasons behind the public’s responses. They also extracted five themes which were about “staying positive amid the storm”, “fear and concern”, “panic buying and hoarding”, “reality and expectations about the situation” and “worries about the future”.	
Mackey, et al. [23]		COVID-19		Worldwide		4 million (3 March 2020 to 20 March 2020)		Tweets were clustered into five main thematic categories: symptom reporting concurrent with lack of testing, discussion of recovery, conversations about first and second-hand reports of symptoms, confirmation of negative diagnosis, and discussion about recalling symptoms	
Alomari, Ebtesam, et al. [36]		COVID-19		Saudi Arabia		14 million (1 February 2020 to 1 June 2020)		Detected fifteen public concerns and government pandemic measures as well as six macro-concerns (social sustainability, economic sustainability, etc.), and formulated their information-structural, temporal, and spatio-temporal relationships	

This study addresses the research question: *how did people in New Zealand understand and react to the emergence and spread of COVID-19 and subsequent events*? The value of this study lies in the potential to use technology to make sense of the general public’s topics of interest in real time. This information may be valuable for those interested in how people react to emergent events, including researchers, governments, and policy makers.

## 2. Background and related works

Evidence suggests that during a crisis, such as COVID-19, individuals use social media platforms such as Facebook, Twitter and Instagram to make sense of the situation by communicating and discussing their experience and opinions [6–8]. Mirbabaie, et al. [9] suggest that information from social media fills the information gap often experienced from more traditional information or media sources. This has been apparent during recent events such as terrorist attacks, floods, bushfires and similar catastrophes [6, 9]. COVID-19 has also seen individuals and communities increasingly relying on social media platforms for communications in an attempt to make sense of the pandemic [1, 10].

During times of crisis, governments are often challenged beyond their capabilities. The measures they take in managing the crisis are crucial [5]. Unlike terror attacks or natural disasters, COVID-19 is unique among crisis events due to its prolonged timeline with no surety of closure, and with potentially greater impact on individuals and society. One avenue that may assist governments in handling crises is the information that can be gleaned from social networks. While social media has been criticized for “misinformation, scaremongering or trivialization of a crisis” [9], it is still an excellent source for rich information that can help governments make sense of how people are experiencing the events that are unfolding [9]. Interacting with others through social media helps individuals trying to make sense of events that are new, uncertain, confusing or violent [11, 12]. This information is potentially valuable to governments and policy makers.

Although social media is not the only medium for sensemaking during crisis events, research suggests that social media plays a key role as a communication platform during extreme times [7]. The nature of social media facilitates two-way communication during crisis events in real time [13]. Such participation leads to sensemaking of the crisis within the social media platform [7] and creates user-generated content ripe for analysis [11, 14, 15].

As a result, in the last several years, the use of big data analytics to make sense of the tremendous volume of first-hand user-generated information accumulated on social media is becoming increasingly popular [e.g., 3, 16, 17–19]. Amongst social media platforms, Twitter has been the platform of most interest to researchers [20]. The reasons for this are twofold: (i) ease of access to data for research, and (ii) the social and instantaneous nature of tweets [21]. As alluded to above, Twitter allows messages with a maximum length of just 280 characters to be shared, which may result in a more direct ‘sensing’ of the environment.

As Table 1 depicts, especially after the emergence of COVID-19, several studies have examined how people in different societies, or around the world, have reacted to COVID-19 matters on Twitter. The focus, approach, and scope of these studies vary depending on the objectives and interests of the researchers. Some look into worldwide matters to uncover common patterns and/or address specific questions on a global scale. For example, Xue, et al. [3] examined around 2 million tweets related to COVID-19 (fetched through 19 trending hashtags) and identified 11 common COVID-related topics being discussed internationally. Lyu and Luli [22] analyzed over half a million tweets to make sense of public perceptions of social distancing through organic, large-scale discussion on Twitter. Mackey, et al. [23] investigated 4 million tweets worldwide to detect and characterize user-generated conversations that could be associated with COVID-related symptoms, experiences with access to testing, and mentions of disease recovery. In parallel to worldwide studies, another stream of works zero in on specific countries and regions to understand and make sense of their particular response and reaction to the COVID-19 pandemic, as reflected in Twitter conversations. For instance, the focus of Lyu and Luli [22] and Li et al. [24] was on the United States where the former extracted the topics and overarching themes emerging from the public COVID-related tweet discussion about the Centers for Disease Control and Prevention, and the latter used Twitter posts to detect stress symptoms related to COVID-19 in the US. Others have studied Italy [17], Brazil [25], China [26], the Arab region [27], and so on (See Table 1). Given the domestic focus of these studies, many have investigated languages other than English, for example Alshalan, et al. [27] examined around 14 million Arabic tweets and identified fifteen public concerns and government pandemic measures as well as six macro-concerns. Our study contributes to this growing body of research (i.e., the stream of research focused on studying nations’ and regions’ response to COVID-19) through examining the themes and trends of Twitter conversations by New Zealanders–a nation successful in battling COVID-19. To the best of our knowledge, this study is the first attempt to uncover the COVID-related topics of discussion–on Twitter–amongst New Zealanders.

## 3. Research design

### 3.1. Context: COVID-19 in New Zealand

New Zealand (NZ) undertook a ‘go early and go hard’ approach to tackle COVID-19. In their strategy to combat COVID-19, the Ministry of Health NZ identified four pillars of elimination as: (i) border controls, (ii) robust case detection and surveillance, (iii) effective contact tracing and quarantine, and (iv) strong community support and control measures [37]. Three days following the WHO’s announcement of COVID-19 as a public health emergency, on 30^th^ of January 2020, the NZ government banned foreigners from/via China and implemented a 14-day self-isolation period for New Zealanders travelling from or through China. The first case of COVID-19 landed in New Zealand on the 28^th^ of February 2020, and the travel ban for foreigners was extended to more countries. New Zealand’s Prime Minister, Jacinda Ardern, announced a four-level alert system on 21^st^ March, identifying NZ’s status on the day as level 2. On 23^rd^ March, with 102 cases reported across the country, the first community transition was detected, and the government lifted the alert level from 2 to 3 and then to 4 (total lockdown, i.e., the maximum restriction) on 25^th^ March [38]. At alert level 4 people were instructed to stay home. All educational institutions and businesses (except limited essential services) were closed [39]. The strict lockdown was successful in containing the virus. The country reverted to alert level 3 on 27^th^ April, to level 2 on 14^th^ May, and eventually dropped to level 1 (no restrictions except border closure to foreigners) on 8 June 2020. After enjoying over 100 days with no community transition, a second wave of community transmission hit the country from an unknown source on 12^th^ August 2020. The government immediately enforced alert level 3 for Auckland and level 2 for the rest of the country.

### 3.2. Data collection and pre-processing

Using the Twitter API (Application Programming Interface) The Twitter developer API was used in this study. Access to data was in accordance with the Twitter terms of service. According to the Twitter Developer Agreement and Policy (https://developer.twitter.com/en/developer-terms/agreement-and-policy), consent and permissions section, no consent from the account owners is required should the tweets be used (e.g., read or analyzed) with no modifications (which was the case in our study). Moreover, we only used “get” type actions which do not require seeking permissions. Further, we only used public accounts (not protected and blocked accounts given that accessing protected and blocked accounts with the developer API is not permitted)., we collected tweets posted by New Zealanders from 29 December 2019 (announcement of COVID-19 in the world) to 19 August 2020. Geotag information was used to identify New Zealand accounts. We searched for those accounts whose geotag information fell within 1200 km of the longitude and latitude of Wellington (the capital of New Zealand) in each direction (which covers all of New Zealand). Any Twitter users within this geographic boundary who were active between 3 and 11 August 2020 (one week) were selected. This resulted in identifying 187,210 unique accounts. Then, several filtering steps were applied to ensure the quality of selection process. First, protected accounts (whose tweets cannot be collected using the API) and the bot accounts were dropped which left 167,185 unique users. Next, outlier analysis was conducted with the aid of SPSS software. Removing outliers left 108,293 accounts nationwide for the analysis [40]. Then around 20% of the accounts were randomly selected and the tweets they had posted from 29 December 2019 to 19 August 2020 were collected. We then queried the collected tweets with a range of keywords and hashtags associated with COVID-19 as listed in S1 Appendix. This eventually resulted in a dataset of 405,427 COVID-related tweets posted by New Zealand residents. Before conducting topic modeling, data pre-processing was performed to ensure the quality of our topic modeling analysis, which involved data cleansing, filtering out the stop words, converting the whole text to lower case, tokenizing, and stemming [41]. We elected to remove the retweets as too many instances of the same post can skew the clusters without necessarily increasing any discriminatory value. Because our data was collected randomly, the original tweets of many retweets were absent in our dataset. Therefore, we retained the first instance of a retweet if the original tweet was not included in our dataset (n = 251,461).

**Fig 1:**
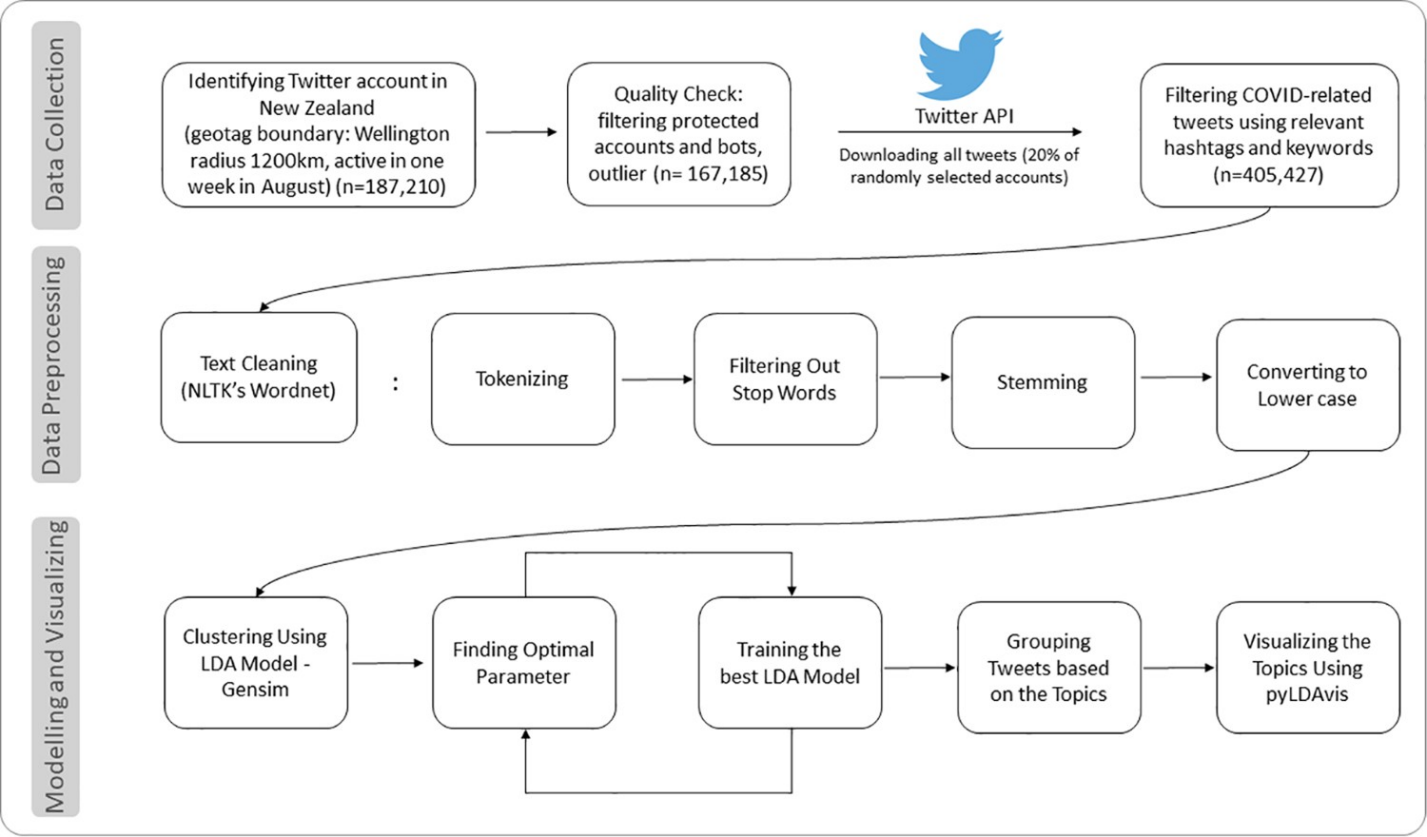
The process for data collection, pre-processing, and analysis.

We used Python for programming the topic modeling process. NLTK (www.nltk.org) [42] was used for tokenizing and stemming. In tokenizing, the meaningful parts of the tokens (i.e., words) were separated from punctuation marks, spaces, emojis and other symbols [41]. We however retained ‘@’ to be able to distinguish ‘mentions’ from the main body of the tweets. Tokens with a number of characters below the minimum threshold of 3 were also removed [43]. All text was converted to lowercase. In stemming, the words were reduced to their grammatical root and converted to lowercase [41]. Fig 1 visualizes the collection, pre-processing, and analysis of data.

### 3.3. Topic modeling

Using computerized analytics to make sense of large volumes of unstructured text is at the heart of academic endeavor where manual analysis is not possible due to the limitations of human data processing [9, 44, 45]. In this domain, *topic modeling* is a popular technique for the purpose of sensemaking [43, 46, 47] and refers to "a collection of algorithmic approaches that seek to find structural patterns within a collection of text documents, producing groupings of words that represent the core themes present across a corpus” [44, p. 1250]. To undertake topic modeling in the present research, we used Latent Dirichlet Allocation (LDA) [48], a popular algorithm that is considered to be the de facto standard for topic modeling [44, 49]. Table 2 shows a brief list of prior studies that have used LDA to detect common topics in large bodies of text data, across various contexts and use cases. LDA is a probabilistic Bayesian network model which characterizes each document (tweet in our case) with a range of keywords and then forms topics based on co-occurrence of keywords in the same document with a certain probability. This means that a topic emerges when a cohort of keywords appear often together in a certain number of documents [47].

**Table 2:** Sample of studies that used LDA.

Study	Context	Data/platform	LDA Outcome
Alomari, Ebtesam, et al [36]	COVID-19	14 million Tweets	Identifying government pandemic measures and public concerns
Mortensona and Vidgen [44]	Literature review	3,386 research articles	Analyzing the literature on the technology acceptance model
Stokes, et al [50]	COVID-19	94,467 related comments on Reddit	Identifying the daily changes in the frequency of topics of discussion across COVID-19-related comments on an online public forum (Reddit)
Wang, et al [26]	COVID-19	203,191 Sina Weibo Microblogging posts	Identifying the most common topics posted by users and performing user behavior analysis on the topics
Rortais, et al [51]	Detecting food fraud incidents	2276 news articles	LDA was applied on a media corpus in order to detect rapidly specific food fraud incidents in the media (i.e. on the Europe Media Monitor Medical Information System)

For LDA analysis, we used Gensim (www.pypi.org/project/gensim/) in the Python environment. As with most exploratory statistical methods (such as factor analysis or clustering), the topic modeling algorithms are not able to label the topic. Labeling must be done by humans based on the content of the topics. Visualizations of the topics in a graphical form is an effective means to assist people with interpreting and labeling the clusters. In the present research, we used LDAVis [52] to help us make sense of the topics and label them. LDAVis is a powerful web-based interactive program that provides a graphical overview of the topics by showing them in the form of distinct circles. It also shows the terms that are most highly associated with each topic as well as the degree of relevance of each term to different topics (see Fig 3). It allows users to look into each individual topic while at the same time keeping the bigger picture of the entire topic model in view [44].

One of the limitations of topic modeling is that the optimum number of topics (typically denoted by *k*) does not emerge from the LDA analysis itself; rather it is something for the researcher to determine in advance and feed to the algorithm as an input variable [43, 48]. Determining this number is a rich and popular area of research but still in development with no “road-tested” answer [44, 46]. A common alternative is experimenting to find “a trade-off between information loss and information overload”, that is, to form “enough topics within each concept to uncover the internal variability, without having an excessive number of topics that would create noise and hamper comparability among concepts” [43, p 719]. After experimenting with *k* between 5 and 10 and analyzing the output, we eventually found *k* = 5 to be the most interpretable topic model. This number is close to default options suggested in the literature, for example by Knutas, et al. [53] and D’Amato, et al. [43]. The findings of the topic modeling are presented and discussed in the following sections.

## 4. Results

**Fig 2:**
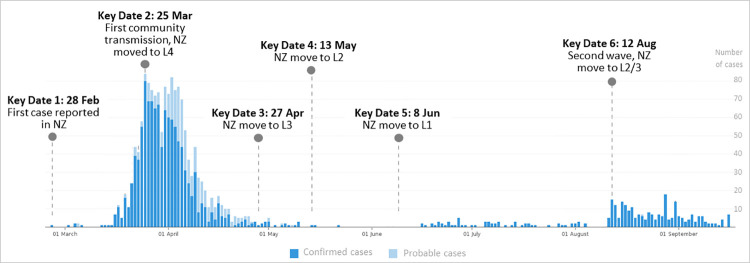
Key dates in the NZ COVID-19 experience (case stats adopted from *NZ COVID-19 dashboard*, Ministry of Health: https://nzcoviddashboard.esr.cri.nz/).

To make sense of COVID-19 tweets posted by the New Zealand population, we conducted topic modeling analysis (using LDA and LDAVis) on our entire dataset as well as on the tweets published around six key dates (KD1 to KD6) when a major COVID event occurred in NZ, from the start of pandemic in late December 2019 until 19 August 2020. The key dates are: (i) 28 February 2020 (KD1) when the first case was formally reported, (ii) 25 March 2020 (KD2) when the first community transition took place and the country moved to alert level 4, a strict nation-wide lockdown, (iii) 27 April 2020 (KD3), stepping down to alert level 3, (iv) 3 May 2020 (KD4), stepping down to alert level 2, (v) 8 June 2020 (KD5), stepping down to alert level 1, and (vi) 12 August 2020 (KD6) when a second wave hit the country and the government enforced alert level 3 for Auckland and level 2 for the rest of the country. The timeline and the COVID case distribution are demonstrated in Fig 2 (note the situation was still evolving at the time of writing this paper. After we finished our data collection [19 August 2020], and during data analysis, Auckland dropped to level 2 on 30 August, but that time stamp is not covered in this study).

As mentioned in the method section, we programmed LDA to extract five topics. Each topic is characterized by a set of terms. The LDAVis package highlights the top 30 most salient terms. Through adjusting a relevancy parameter (0 ≤ λ ≤ 1), LDAVis provides the option to reveal the terms that are more common to the entire corpus (higher λ values) or more specific to the cluster under examination (lower λ) [52]. Given that we had a range of context terms such as coronavirus and COVID-19 with limited discriminatory value, we found that lower values for λ (between 0 and 0.2) returned more interpretable topics.

**Fig 3:**
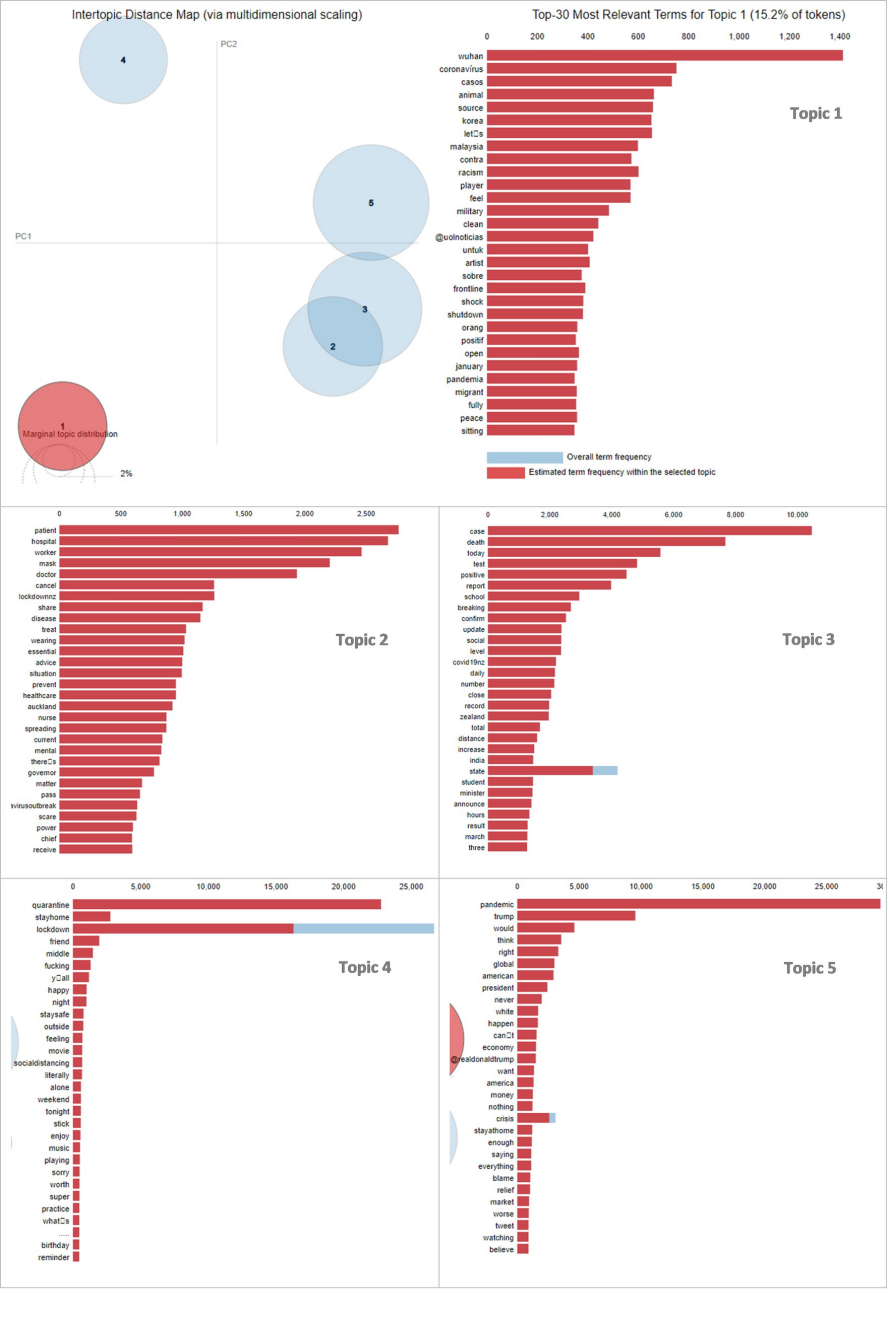
LDA topic modeling for entire dataset including top 30 salient terms for each cluster (λ≈0.2).

To label the clusters, two authors of the paper examined and discussed the topic models (for the entire data set and for each of KD1 to KD6) in several iterations and labeled the clusters based on the salient keywords highlighted by the LDA algorithm and LDAVis visualizations. The labels were then discussed with the other four authors until consensus between the whole team was achieved. The results are presented in Table 3 for the entire dataset, and Tables A1 –A6 in S2 Appendix for KD1 to KD6, in which the topic labels, most important terms (or tokens), and the percentage of the terms comprising each particular topic are reported. As an example, the LDAVis visualization for the whole dataset is shown in Fig 3. The same visualizations were produced for KD1 to KD6. Readers are referred to the online supplementary material of this paper on the journal website or alternatively on [*this link–removed for blind review process*] for an interactive online version of extracted topic models. In the LDAVis output, the size of the circles reflects the proportion of each topic within the whole corpus and the location of the circles represents the semantic distance between the topics based on the co-occurrence of the terms [43, 52].

**Table 3:** Topics extracted from entire tweets dataset.

No.	Topic label	Important selected terms (see all words in supplementary online material)	% Tokens
T1	Worldwide matters	Wuhan, coronavirus, animal, source, Korea, Malaysia, racism, military, frontline, shutdown, pandemic, migrant, January, peace	15.2%
T2	Health matters in NZ	Patient, hospital, mask, doctor, disease, treat, wearing, essential, prevent, healthcare, nurse, spreading, mental, advice, lockdown, nz government coronavirus, outbreak, scare, Auckland	19.1%
T3	Government response and measures	Case, death, test, positive, report, breaking, confirm, number, level, record, distance, increase, Zealand, total, minister, announce, result, march	25%
T4	Leisure and entertainment	Friend, happy, night, staysafe, movie, weekend, alone, tonight, enjoy, music, playing, birthday, celebrate, stayhome, quarantine	14.9%
T5	Politics and economy impact	Trump, pandemic, global, America, president, white, economy, money, blame, market, worse, @realdonaldtrump	25.8%

## 5. Discussion

Overall, the outcome of our LDA analysis reveals a number of insights which are helpful for making sense of New Zealanders’ communications since the start of pandemic. Analyzing our whole dataset (all COVID-related tweets since the last days of 2019 until 19 August 2020, n = 405,427) highlighted five clusters, two of which (T1 and T4) stood clearly apart from others, and three (T2, T3, T5) that overlapped to some extent (Fig 3 and Table 1). As the keywords in Table 1 imply, Topic 1 appears to focus on the world-wide matters associated with COVID-19 (e.g., the source of the virus from an animal market in Wuhan). Topic 3 refers to a range of keywords which seem to be linked to the impact of COVID-19 on New Zealand and the response to it (e.g., case, death, distance, ministry, announce, result). Topic 2 is more specific and is clearly focused around health matters by containing obvious healthcare terms such as doctor, patient, hospital, health, mental, and disease. Topic 4 advocates that New Zealanders have not forgotten about leisure and entertainment during the tough time of the pandemic as terms such as music, playing, celebrate, weekend, movie, and birthday form a distinct cluster, although it is smaller (14.9%) than the others. The marginally largest topic of discussion amongst New Zealanders was Topic 5 which implies politics (interestingly mostly around the US, e.g., Trump, president, America, white, blame) and the economy (money, market, economy, worse), comprising 25.8% of the tokens.

**Fig 4:**
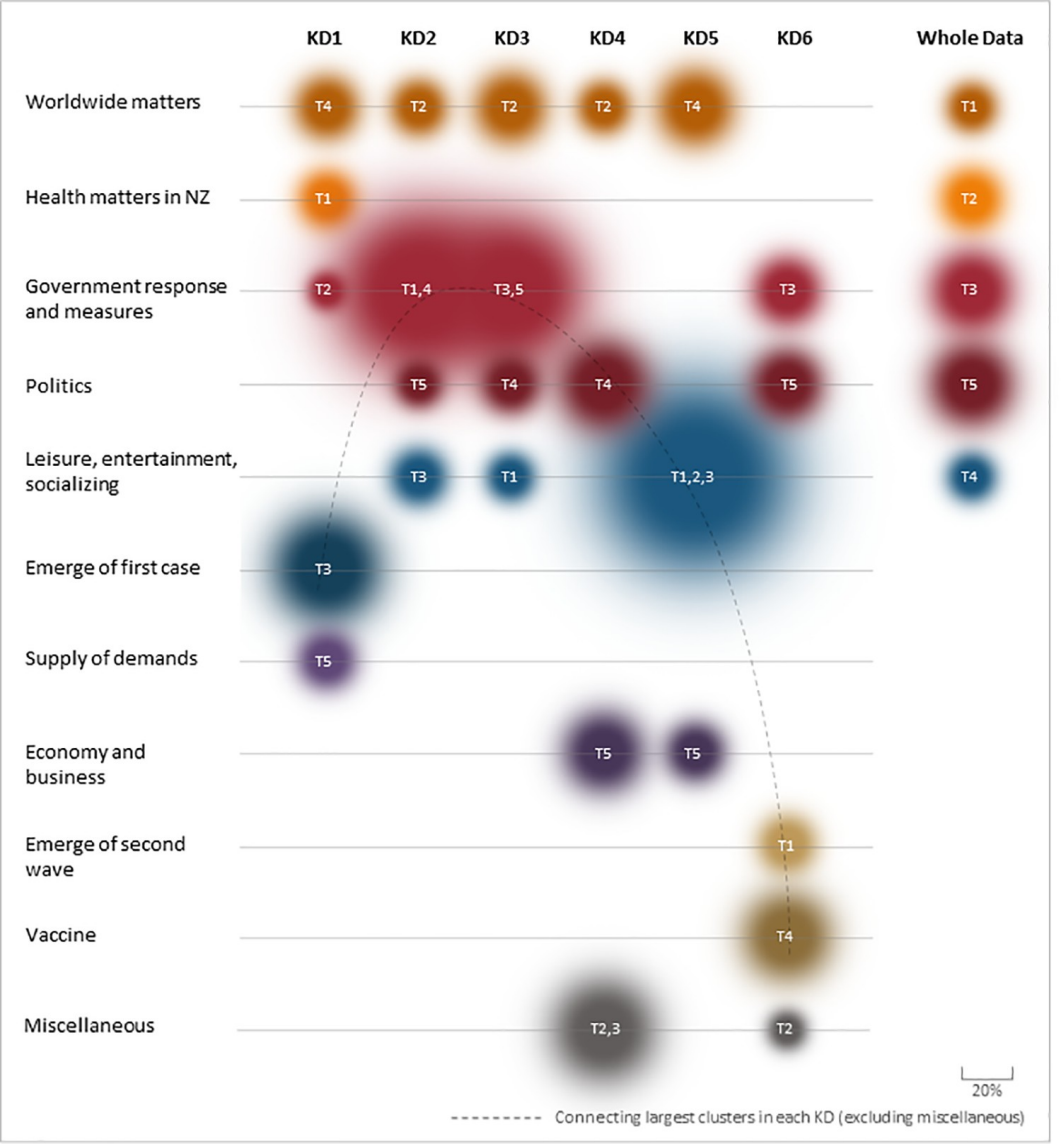
An overview of the topics landscape across the key dates, and overall.

Fig 4 provides a visual representation to foster our understanding and sensemaking of the COVID-19 tweet landscape in New Zealand and its temporal development and evolution over time. The figure was produced using a graphical tool–Adobe Photoshop–based on the outcome of the LDA analysis and our subsequent interpretation of the clusters/topics. The size of the bubbles represents the percentage of tokens in each category as per the numbers in the last column of Table 3 (for whole data) and Tables A1 to A6 in S2 Appendix (for the six key dates). Some topic categories were quite similar or identical, thus we merged them together into one bubble for a more meaningful and informative representation in Fig 4‘s visualization, for example, T1 and T4 in KD2 both refer to government response and measures (see Table A2 in S2 Appendix), and were thus merged. It is apparent that the international elements of the COVID-19 event have been a constant topic of concern in NZ tweets as implied by frequent references to names of many countries and recognizable individuals (e.g., Boris Johnson), or international institutions (e.g., IFRS: the International Federation of the Red Cross). In some clusters, a more specific focus is evident. For example, in T4 for KD1 (see Table A1 in S2 Appendix), it appears that people are reacting to the dishonesty, blaming, conflicts, and perhaps conspiracy theories around the world in relation to COVID-19 (terms: Chinese, Wuhan, lying, TrumpIsIdiot, conspiracy, psycho, insanity).

Interestingly, United States politics is significantly represented in the Twitter communications of the New Zealanders. In four out of the six key dates, we observed explicit clusters talking about the USA with the frequent emergence of the names of key politicians (Trump, Obama, Biden, etc.). Of particular note, Topic 4 in KD3 (the tweets around 27 April 2020, see Table A3 in S2 Appendix) clearly advocate a backlash to President Trump’s daily briefing, specifically on 23^rd^ of April when many perceived that Donald Trump was suggesting the injection of disinfectants into people to fight the coronavirus (terms: Donald, disinfectant, inject).

The data suggest that some topics are almost consistently present in New Zealanders’ contributions to Twitter over time–including worldwide matters, politics, and government responses and measures–but at the same time, prominent local events are also well reflected in people’s communications and posts. We noted that on KD1, one specific cluster (T3) seems to be quite focused around the emergence of the first confirmed case in New Zealand. Likewise, when the second wave hit in August, an obvious cluster (T1) was formed around the post related to the re-emergence of new cases in Auckland and the concerns and anxiety surrounding it (terms: test, positive, symptoms, high, negative, panic, critical, lockdownnz, etc.) (Table A6 in S2 Appendix). We also observed a cluster (T4) in KD6 (12 August 2020) with a range of keywords associated with vaccines (e.g., vaccine, dose, potential, Russia) (Table A6 in S2 Appendix). This cluster meaningfully coincided with an announcement from the Russian government on 11^th^ of August when they formally released and used a vaccine for COVID-19, although the safety of the vaccine was widely questioned by other countries and international authorities at the time [54].

Overall, from what can be seen in the data, it seems New Zealanders’ communications, dialogues, and discussions–at least those expressed on Twitter–have been shifting in response to the country’s up and down experience with COVID-19. The dashed line in Fig 4, which connects the largest topic cluster of each key date, in a broad sense reflects the general movement of New Zealanders’ concerns and discussions during the first several months of the pandemic. In the beginning, when the coronavirus was detected in New Zealand for the first time in late February 2020, it triggered a significant level of concerned discussion in New Zealand (T3 in KD1). New Zealand managed to contain the virus and avoided community transition for about a month (via isolating passengers in government-managed quarantine facilities and extensive contact tracing) but eventually the virus broke through and the first community transition occurred in late March, putting the country in total lockdown. In the early days of lockdown when the country was experiencing severe restrictions, high levels of anxiety, and many unknowns, the tweet landscape was mostly framed around the immediate, direct, and specific measures and actions required to contain the disease and to fight the virus. In KD2 (alert level 4) and KD3 (alert level 3), the biggest proportion of tweets was linked to the country’s response to the virus and the government measures and actions (salient keywords such as isolation, stayhome, social, distance, test, symptom, Jacinda, Ardern, etc.). But over time and as the situation seemed to come under control (relatively fast in New Zealand), the tensions seemed to ease and people increasingly talked about entertainment, leisure and social aspects of life, even as these social aspects were redefined within the COVID-governed world. In a sense, a “new normal” reality had been recognized and accepted. In KD5, when New Zealand was almost free from restrictions (except border closure), we observed three clusters implying stress relief and enjoyment (T1), entertainment and leisure (T3) and socializing (T2) which together comprise 58.5% of the keywords. When the second wave of COVID-19 (and subsequent lockdown) hit New Zealand in August 2020 (KD6), a considerable volume of dialogs took place again around government measures (T3 in KD3) (after an absence of concentration on this topic in KD4 and 5). Interestingly, however, the biggest topic of tweets in KD6 (though by a slight margin) was formed around vaccines. That was a sensible finding given the increasing attention to vaccines at the time, especially the formal announcement of the vaccine rollout in Russia by the government on 11 August 2020 (despite international concerns about its safety and effectiveness at the time) [54]. All in all, our findings suggest that Twitter offers a sensible and meaningful basis for understanding and making sense of how people react to critically changing circumstances in pandemic outbreaks and chaotic conditions, over time and in real time.

## 6. Conclusion, limitations and future research directions

The global health crisis of COVID-19 has been disrupting people’s daily lives, affecting societies, destroying businesses, challenging governments, and in general causing physical and psychological distress. During this crisis, people have been using social media to communicate information, voice their opinions, and engage in discussions to make sense of the situation. In this paper, we analyzed tweets by New Zealanders from the time COVID-19 started in the last days of 2019 until 19 August 2020. Through the use of topic modelling, we have shown how New Zealanders, as citizens of a country successful in tackling COVID-19, reacted to COVID-related events through the topics they discussed on Twitter.

Our analysis of Twitter data shows changes in New Zealander’s concern over COVID-19 events and gives us some insight into how they are making sense of events. This analysis is limited to topic modelling. Other forms of analysis, such as social network analysis (SNA) [9] and sentiment analysis [40], might yield further insights into how the cognitive process fundamental to sensemaking occurs. Another possible future research avenue is to continue developing topic modeling as well as sentiment and social network analysis into live business intelligence-like dashboards that could help identify the key challenges communities are facing and act as a possible source for governments to make informed decisions around COVID-19 kinds of events.

## Supporting information

S1 Data Interactive online version of extracted topic models. Available on Journal website.
(ZIP)

S1 Appendix Search terms to identify COVID-19 related tweets. (DOCX)

S2 Appendix Topic modelling for KD1 to KD6. (DOCX)
