# Supplementary material for: Making sense of COVID-19 over time in New Zealand: Assessing the public conversation using Twitter
Source: PLoS One. 2021 Dec 15;16(12):e0259882. doi: 10.1371/journal.pone.0259882 (PMC8673617; doi:10.1371/journal.pone.0259882)
Supplement: S1 Appendix — (DOCX) [file pone.0259882.s002.docx]

# S1 Appendix: Search terms to identify COVID-19 related tweets

covid19, covid-19, “covid 19”, coronavirus, #covid19nz, #nzcovid19, #coronavirus, #coronavirusnz, #nzcoronavirus, #covid19, #stayathome, #StayAtHomeNZ, #NZStayAtHome, #StayHomeNZ, #NZStayHome, #StayHome, #staysafe, #nzstaysafe, #staysafenz, #bekind, #bekindNZ, #NZbekind, #lockdown, #nzlockdown, #lockdownnz, #NewZealandlockdown, #selfisolationnz, #nzselfisolation, #FlattenTheCurve, #FlattenTheCurveNZ, #NZFlattenTheCurve, #Stillstayinghome, #StillstayinghomeNZ, #NZStillstayinghome, #stillsavinglives, #stillsavinglivesNZ, #NZstillsavinglives, #teamof5million, #teamof5millionNZ, #NZteamof5million, #rāhui, #UniteAgainstCovid19, #NZUniteAgainstCovid19, #UniteAgainstCovid19NZ, #pandemic, #lovelocal, #NZhellhole, #Quarantine, #NZQuarantine, #QuarantineNZ, #managedisolation, #managedisolationNZ, #NZmanagedisolation, #Contacttracing, #ContacttracingNZ, #NZContacttracing, #communitytransmission, #bordercontrol, #bordercontrolNZ, #NZbordercontrol, #borderclosed, #borderclosedNZ, #NZborderclosed, #wagesubsidy, #wagesubsidyNZ, #NZwagesubsidy, #alertlevels, #nzalertlevels, #alertlevelsnz, #socialdistancing, #NZsocialdistancing, #socialdistancingNZ, #physicaldistancing, #NZphysicaldistancing, #physicaldistancingNZ, #maskup#NZmaskup, #maskupNZ, #wearamask, #NZwearamask, #wearamaskNZ, #antimask, #wearamaskNZ, #NZwearamask, #antimaskers, #NZantimaskers, #antimaskersNZ
