# Supplementary material for: Making sense of COVID-19 over time in New Zealand: Assessing the public conversation using Twitter
Source: PLoS One. 2021 Dec 15;16(12):e0259882. doi: 10.1371/journal.pone.0259882 (PMC8673617; doi:10.1371/journal.pone.0259882)
Supplement: S2 Appendix — (DOCX) [file pone.0259882.s003.docx]

# S2 Appendix: Topic Modelling for KD1 to KD6

**Table A1. Topics extracted for Key Date 1: 28 February (reporting first case in NZ)**

| No. | Topic label | Important terms | % Tokens |
| --- | --- | --- | --- |
| T1 | Health concerns | Chance infect kill fight happen support disaster infectious catching staff | 17.7% |
| T2 | Government response and measures | Government federal schedule mission update thread prevent protect achieve specialist general isolation repurposing stations hands testing | 11.7% |
| T3 | Emergence of first case | First case Zealand found recent start country symptom cough impose | 33.2% |
| T4 | Worldwide dishonesty and chaos | Chinese, Wuhan, lying, trumpisidiot conspiracy psyche insanity Russian bekind Bloomberg trumpliesaboutcoronavirus | 19.3% |
| T5 | Supply of demands | Concern demand supermarket customer explode stockpiling wholesaler monitor close disappointment safety | 18.1% |

**Table A2. Topics extracted for Key Date 2: 25 March (first community transition. NZ moved to Alert Level 4)**

| No. | Topic label | Important terms | % Tokens |
| --- | --- | --- | --- |
| T1 | Government response and measures | Test positive symptoms hands isolate alert disease Jacinda prime minister expert recover border | 27.6% |
| T2 | Worldwide matters | Boris Johnson Philippines Canada ministry death confirm passenger @norbertelekes record quarantinelife setting | 17.1% |
| T3 | Entertainment, leisure | Enjoy local party happening Netflix Instagram quarantinewatchparty girl picture nationwide | 17.5% |
| T4 | Government response and measures | Social distance stayhomenz instead living remain claim donate vulnerable victim guideline battle dying trust decision victim office | 23.5% |
| T5 | Politics | Trump America survive worldwide earth protection surpass | 14.3% |

**Table A3. Topics extracted for Key Date 3: 27 April (NZ moved to Alert Level 3)**

| No. | Topic label | Important terms | % Tokens |
| --- | --- | --- | --- |
| T1 | Leisure, entertainment | AvengersAssemble QuarantineWatchParty @comicbook freedom @brandondavisbd (king of quarantine watch party) connect attentions crowd | 15% |
| T2 | Worldwide matters (International widespread) | Wuhan Africa million billion confirm influenzas quickly positive antibody approve surpass warning social distance mask @bbcworld | 21.5% |
| T3 | Government response and measures (lift of restrictions) | Local lift contact tracing nursing takeaway adult blame panic prepare saving except tracing alert celebrate | 21.4% |
| T4 | Politics (America) | Donald disinfectant inject supply service coffee bottle | 17.9% |
| T5 | Government response and measures | Police minister lockdown quarantine Zealand Jacinda Ardern advice watch ease company excuse | 24.1% |

**Table A4. Topics extracted for Key Date 4: 13 May (NZ moved to Alert Level 2)**

| No. | Topic label | Important terms | % Tokens |
| --- | --- | --- | --- |
| T1 | Worldwide matters (Recovery/health) | Rapid antibody immunity recovery reaction strict survivor Spain France continent @francescorocca @ifrc (International Federation of Red Cross) @francescorocca (president of) @whoafro (WHO Africa), Africa, Vietnam | 16.2% |
| T2 | Miscellaneous | Story local effort move street living likely success piece trust venture apologies investor | 18.5% |
| T3 | Miscellaneous | Trading biotech potential @stayhomeshopsuperbalist hopefully comprise Aussie | 12.8% |
| T4 | Political (America) | Obama Biden indigenous anxiety blame advise destroy meeting America Obamagate | 27.8% |
| T5 | Economy and business | Business online money hours worse worst estimate raise help online physical library leave | 24.6% |

**Table A5. Topics extracted for Key Date 5: 8 June (NZ moved to Alert Level 1)**

| No. | Topic label | Important terms | % Tokens |
| --- | --- | --- | --- |
| T1 | Stress relief and enjoyment | stressrelief easing enjoy stress relief artist artwork perspective kitten journalist college catsofthequarantine | 16.3% |
| T2 | Socialising | visitor young concert program hospitalisation mental safety trial rescue volunteer university mother sharing | 18.5% |
| T3 | Entertainment leisure | Movie playing tonight dance watching charity kitchen pupil | 23.7% |
| T4 | Worldwide matters (international crisis) | Stupid shock suicide effect European England @rallysweden African campaign turning ongoing eradicate lose | 23.2% |
| T5 | Economy | Vote register illegal lawsuit lobby activity unemployed essential funding Amazon voteblue hypocrisy healthcare | 18.3% |

**Table A6. Topics extracted for Key Date 6: 12 August (second ware of outbreak. Auckland moved to Alert Level 3, rest of country to level 2)**

| No. | Topic label | Important terms | % Tokens |
| --- | --- | --- | --- |
| T1 | Consequences of COVID re-emergence | Test positive symptoms high negative department breach homeless panic critical lockdownnz responsibility legal officerhungryemployee status | 17.8% |
| T2 | Miscellaneous | Design summer print disabilityvisibility hearing piece website September | 12.2% |
| T3 | Government response and measures | Level confirm prime cluster remain notice alert stage strategy freedom review reduce teacher adult reopen expect | 21.1% |
| T4 | Vaccine | Vaccine dose Russia potential Jacinda Ardern France Sweden Victoria chance normal conspiracy theory Australia transmission appear | 27% |
| T5 | Politics | Biden Obama Russian history consequence feeling mental sense agree shock director | 21.9% |
